# Medication overuse and drug addiction: a narrative review from addiction perspective

**DOI:** 10.1186/s10194-021-01224-8

**Published:** 2021-04-28

**Authors:** Tatiane Teru Takahashi, Raffaele Ornello, Giuseppe Quatrosi, Angelo Torrente, Maria Albanese, Simone Vigneri, Martina Guglielmetti, Cristiano Maria De Marco, Camille Dutordoir, Enrico Colangeli, Matteo Fuccaro, Davide Di Lenola, Valerio Spuntarelli, Laura Pilati, Salvatore Di Marco, Annelies Van Dycke, Ramla Abuukar Abdullahi, Antoinette Maassen van den Brink, Paolo Martelletti, on behalf of the European Headache Federation School of Advanced Studies (EHF AS)

**Affiliations:** 1 Headache Research, Wolfson CARD, Institute of Psychiatry, Psychology & Neuroscience, King’s College London, 20 Newcomen St, London, SE1 1YR UK; 2 Present address: Medicines Discovery Catapult, Block 35, Mereside, Alderley Park, Cheshire, SK10 4TG UK; 3 Neuroscience Section, Department of Applied Clinical Sciences and Biotechnology, University of L’Aquila, Via Vetoio 1, Coppito, 67100 L’Aquila, Italy; 4 Department of Health Promotion, Mother and Child Care, Internal Medicine and Medical Specialties (PROMISE), University of Palermo, Piazza delle Cliniche 2, 90127 Palermo, Italy; 5 Department of Biomedicine, Neurosciences and Diagnostic (Bi.N.D.), University of Palermo, Via del Vespro 129, 90127 Palermo, Italy; 6 Department of Systems Medicine, University of Rome “Tor Vergata”; Neurology Unit, “Tor Vergata” Hospital, Viale Oxford, 81, 00133 Rome, Italy; 7 Department of Experimental Biomedicine and Clinical Neurosciences, University of Palermo, Via del Vespro, 129, 90127 Palermo, Italy; 8 Pain Medicine Unit, Santa Maria Maddalena Hospital, Occhiobello, Italy; 9 Regional Headache Referral Center, Sant’Andrea Hospital, Via di Grottarossa 1039, 00189 Rome, Italy; 10 Department of Medical, Surgical and Experimental Sciences, University of Sassari, Piazza Università, 21, 07100 Sassari, Italy; 11 Department of Neurology, University Hospital Ghent, Corneel Heymanslaan 10, 9000 Ghent, Belgium; 12 Department of Neurology, Conegliano Hospital, Via Brigata Bisagno, 2, 31015 Conegliano, Italy; 13 Department of Medico-Surgical Sciences and Biotechnologies, “Sapienza” University of Rome, Polo Pontino, Viale XXIV Maggio 7, 04100 Latina, Italy; 14 Division of Pharmacology, Department of Internal Medicine, Erasmus MC University Medical Centre Rotterdam, Wytemaweg 80, 3015 CN Rotterdam, The Netherlands; 15 Headache Centre, Guy’s and St Thomas NHS Trust, London, UK; 16 Department of Clinical and Molecular Medicine, Sapienza University of Rome, Via di Grottarossa 1039, 00189 Rome, Italy

**Keywords:** Drug abuse, Dependence, Migraine, Substance abuse, Vulnerability

## Abstract

Chronic headache is particularly prevalent in migraineurs and it can progress to a condition known as medication overuse headache (MOH). MOH is a secondary headache caused by overuse of analgesics or other medications such as triptans to abort acute migraine attacks. The worsening of headache symptoms associated with medication overuse (MO) generally ameliorates following interruption of regular medication use, although the primary headache symptoms remain unaffected. MO patients may also develop certain behaviors such as ritualized drug administration, psychological drug attachment, and withdrawal symptoms that have been suggested to correlate with drug addiction. Although several reviews have been performed on this topic, to the authors best knowledge none of them have examined this topic from the addiction point of view. Therefore, we aimed to identify features in MO and drug addiction that may correlate. We initiate the review by introducing the classes of analgesics and medications that can cause MOH and those with high risk to produce MO. We further compare differences between sensitization resulting from MO and from drug addiction, the neuronal pathways that may be involved, and the genetic susceptibility that may overlap between the two conditions. Finally, ICHD recommendations to treat MOH will be provided herein.

## Background

Headache is one of the most common neurological disorders with estimated 3 billion people worldwide suffering with some type of headache disorder [1]. Most sufferers are individuals at their most productive ages [2] and thus, preventing the development of secondary disorders as well as finding novel treatments especially for those suffering with chronic headache are important to maintain workforce productivity and quality of life.

When headache frequency occurs for ≥15 days for over 3 consecutive months it fulfills the criteria for chronic headache as defined by International Classification of Headache Disorders (ICHD-3) [3]. Patients diagnosed with chronic headache and mostly chronic migraine (CM) often have a long history of unsuccessful preventive treatments in addition to a high incidence of comorbidities [4, 5] . Causes of headache chronification are not fully understood but in some patients, it has been linked to overuse of medications to abort the headache. In fact, overuse of medication has been reported in almost three-quarters of CM patients [6], suggesting that most headache patients may not be receiving a close follow up from their doctors.

The ICHD-3 defines medication overuse headache (MOH) as a secondary headache that develops from the use of (I) triptans, ergotamine, opioids, or combination-analgesics of two or more classes for at least 10 days a month for > 3 months, or (II) non-steroidal anti-inflammatory drugs (NSAIDs) or paracetamol for at least 15 days a month for > 3 months [3]. Women in their 40’s are three to four times more prevalent than men [6, 7], and that proportion fluctuates similar across countries [7].

MOH is often the result of the progression of long-standing chronic headache disorders, and mostly CM [8]. Whether MOH is a secondary headache originated by the condition of medication overuse (MO) or MO is a consequence of chronic headache disorders remains a matter of debate [9]. Therefore, in the present review, we will focus on the condition of medication overuse (MO) irrespective of the underlying headache.

Some risk factors have been associated with the expression of MO [10], including genetic predisposition, low education level, chronic gastrointestinal complaints, smoking, high caffeine intake, lack of physical activity, and psychiatric comorbidities, e.g. depression and anxiety [6]. Other health conditions that entail frequent use of analgesics, such as chronic pain, may also lead to MO [7]. Although it has been previously suggested that MO and addiction may share a common pathway [11], our aim herein is to provide a review from addiction point of view in order to clarify and identify other common features between MO and drug addiction.

## Medications to abort headache attacks

They are generally separated in two classes, (I) *specific medications*, and (II*) non-specific medications* [12]. *Specific medications* include triptans and ergotamine, which are usually prescribed for migraine and cluster headaches. Their anti-migraine effects are mainly by actions on 5-HT_1_ receptors localized in the trigeminovascular system. Specifically, they inhibit the release of peripheral vasoactive peptides, such as substance P, neurokinin A and calcitonin gene-related peptide (CGRP) that lead to nociceptor activation and consequent trigeminal activation or vasoconstriction of meningeal blood vessels [13–15]. *Non-specific medications* comprise of various active compounds with different mechanism of actions. NSAIDs, aspirin, paracetamol, antiemetics, corticosteroids, and opiates can be prescribed to treat headache. In short, NSAIDs, aspirin, and paracetamol have actions on cyclooxygenase (COX) enzymes that convert the free essential fatty acids to prostanoids, which levels are found increased in inflamed tissue [16]. Blocking prostanoid biosynthesis prevents the neurogenic inflammation and the central sensitization of second-order trigeminal nociceptors that mediates allodynia during migraine attacks [16, 17]. Opioids, e.g. butorphanol, codeine, tramadol, and meperidine, bind to opioid receptors - μ (mu), κ (kappa), δ (delta), and nociceptin/orphanin FQ – that are found throughout the central nervous system, including in the brain regions recruited to pain signaling such as periacqueductal grey area (PAG), cerebral cortex, thalamus, nucleus raphe magnus, rostral ventral medulla, spinal cord dorsal horn, and brain stem [18, 19]. At the cellular level, opioids reduce the overall synaptic transmission as well as inhibit the GABAergic signaling in the brain stem, which results in the inhibition of the pain circuit signaling [18].

Specific and non-specific medications can both cause MO. Patients treated with triptans or opioids are more frequently reported with MO at a shorter time than those undertaking treatments with other medications [6, 20]. On average, triptans produce MO in approximately 1.7 years and opioids, in approximately 4.8 years [21]. NSAIDs and paracetamol exhibit the lowest risk for MO as it is less frequently reported [6, 22, 23]. Although triptans might lead to rapid development of MO its withdrawal is short and withdrawal symptoms diminish rapidly. Conversely, withdrawal from opioid overuse is complex and gradual as patients report strong withdrawal symptoms, requiring in-patient supervision in certain cases [6, 24]. In addition, opioids have high potential of abuse and consequently, a high risk to develop addiction when misused or taken for long periods. The United States have been facing a rise in opioid overdose over the past 30 years and the indiscriminate use of opioids is estimated to cause more than 15,000 deaths a year in that country [25–27]. Europe has also seen an increase in opioid prescriptions in recent years [28] and that brings along fears for an imminent opioid misuse in Europe. Nevertheless, opioids prescriptions are fully discouraged in Europe [29].

## MO and drug addiction: the overlapping features

MO and drug addiction initiate from different contexts and reasons. Smoking and alcohol are normally used as recreational drugs, whereas headache patients seek medications in order to alleviate their feeling of pain [30–32]. Nevertheless, the regular use of drugs or other substances can facilitate the acquisition of Pavlovian learning that may instate a habit formation or even lead to the development of an incentive sensitization [33]. Takahashi et al. (2019) have demonstrated that Pavlovian learning and transfer do not correlate with addictive behaviors [34]. However, the drug associated cues might be able to modify how a particular drug that is regularly used is perceived (see Robinson and Berridge, 2008 for review). More details will be given below about this topic.

Drug addiction is a complex mental disorder that compromises all spheres of life, i.e. behavioral, cognitive, social, and emotional. It is a condition that is characterized by recurrent relapses and impaired inhibitory control over the drug taking and seeking [35]. However, the term “addiction” has been excluded from the fifth revision of the Diagnostic and Statistical Manual of Mental Disorders (DSM-5). Instead, the substance use disorder has been adopted to characterize individuals with different levels of the disorder. In this present review, we will refer to ‘addiction’ to individuals showing severe substance use disorder as the International Classification of Diseases (ICD-10) still uses this term.

The development of drug addiction in individuals is associated with risk factors and some risks factors for MO and addiction may overlap. The risk factors for MO are genetic predisposition, low education level, smoking, and psychiatric disorders, mostly depression and anxiety.

Individuals who have a close relative with migraine are highly susceptible to develop migraine, and different gene loci have been linked to migraine susceptibility [36, 37]. In drug addiction, *genetic predisposition* is also considered a risk factor. However, the development of drug addiction is not strictly related to familiar history of the disease.

Another risk factor for MO, the *low education level*, could perhaps be associated with low income [38, 39]. Individuals that are exposed to frequent life threatening/stressing situations, i.e. hunger, violence, moral and physical abuses, especially at younger age, are often also exposed to bad life examples, which conscious or not, it can shape their way of making decisions. For instance, youth criminality and drug abuse are often associated. Several studies have shown that the early the start of drug use, the higher their risk to develop drug addiction. Additionally, other evidence demonstrates patients with MO have lower educational levels compared with those without MO [40], suggesting that socioeconomic factors are risk factor for both addiction and MO.

The other risk factor that may overlap between MO and addiction is some *psychiatric disorders*. In drug addiction, individuals living in a highly pressured environment and who suffer from high anxiety or severe depression often use drugs as an escape. Anxiety and depression are also associated with the development of MO in patients with headache [41, 42]. However, it is not yet clear whether MO patients with ritualized behaviors take medications for comforting reasons or as a matter of habit. More studies are warranted to clarify all those associations. Nevertheless, the risk factors combined might likely account for the alterations in cortical and subcortical regions that underpin the changes in neuro-circuitry of reward, motivation, memory, and judgment [35, 43], which in turn express as a pathology.

In the behavioral aspect, *sensitization* is a feature found in both addiction and MO though expressed differently. In headache sensitization is expressed as allodynia, a feeling of pain that results from an innocuous stimulus such as light touch. Cutaneous allodynia is a marker of central sensitization and it involves actions of pro-nociceptive mediators, increasing nociceptor responses. Cutaneous allodynia is estimated to affect 50–80% of migraineurs and it has been suggested to predict migraine chronification [44]. In drug addiction sensitization is developed after repetitive, often intermittent, administration of substance of abuse, expressed by enhancement of locomotor activity in mice [45], or an increase in activity/energy level, mood, amount of speech, and eye-blink rates in humans [46]. The increase in the drug effects following repeated drug administration reflects the sensitization of the brain mesocorticolimbic systems [33, 45, 47, 48]. Moreover, a sensitization of the brain incentive systems can further engage the motivation for drugs and drug-cues, and that can lead to a pathological ‘wanting’ for drugs [33]. The dopaminergic system plays a role in both locomotor and incentive sensitization in drug addiction [49], and recently, dopamine (DA) has also been demonstrated in cutaneous allodynia [50]. A study evaluating the dynamics of endogenous DA neurotransmission in migraineurs has shown variations in DA receptor density during the migraine attack accompanied by allodynia [51]. Previous studies have also shown that migraineurs are hypersensitive to dopamine agonists [52] and dopamine D_2_ receptor antagonists can reduce both migraine frequency and severity [53]. Those studies have initiated several discussions among researchers with focus of DA actions on premonitory symptoms of migraine. That is fundamentally based on the fact that A11 hypothalamic neurons to trigeminal neurons are the sole source of dopaminergic neurons to the spinal cord [54]. However, the emotional valence of headache chronification should be taken into consideration as an important component in the development of MO. As demonstrated in previous studies with chronic back pain patients, DA can regulate sensory and affective dimensions of pain, and interestingly, the development of chronic pain is influenced by the striatum [55]. The striatum is a brain region strongly modulated by dopaminergic neurotransmission and it is essentially involved in some addictive behaviors, such as habit formation and drug seeking behavior (see [56, 57] for review). Further studies are still warranted to clarify the mechanisms in which dopamine engages in the chronification of migraine, however, there is a strong indication it might contribute to the expression of MO.

According to the incentive-sensitization theory, DA can modulate *wanting* to take the drug that is distinguished from *liking* the drug [58]. In other words, drug addicts can continue *wanting* the drug despite not *liking* it anymore due to lack of rewarding effects developed through pharmacological tolerance. Based on the theory, MO patients may similarly *want* to take analgesics despite the lack of pain relief effects. It is, therefore, a desire to continue administering analgesics that can be translated as psychological attachment. That attachment might motivate the MO patients to ritualistically take analgesics every morning despite lack of benefit or positive outcomes. Further studies are still needed to clarify whether those behaviors could progress to an obsessive behavior.

Based on the ability of DA in changing the perception of pain in chronic pain patients as well as in altering the motivation for reinforcing activities, it is very likely that the psychological attachment to the drug by MO patients is contributed or governed by DA.

Another common feature for both conditions is the relapse after drug withdrawal [59]. The relapse rate for MO patients is estimated to range between 25 and 35% within the first year [60, 61], whereas for drug addiction it can be over 65% in the first year [62, 63]. In drug addiction, factors such as drug-associated cues and/or stress can trigger craving and the drug seeking behavior. For MO, the type of primary headache and the class of the drug overused by the patient, i.e. opioids and barbiturates [64], the baseline headache frequency [65], and the number of previous preventive treatments [66] have all being associated as predictors to relapse. No factor has yet been identified or directly associated with relapse in MO.

**Fig. 1:**
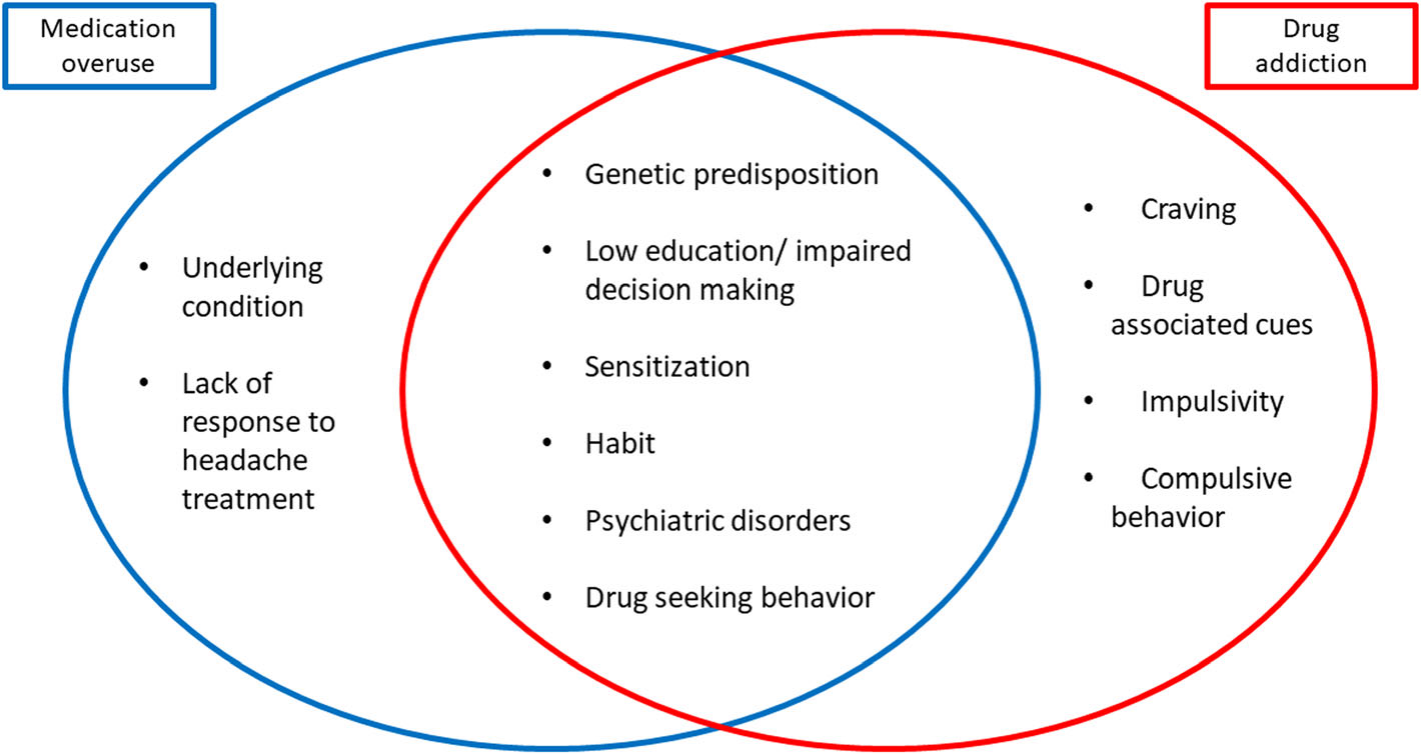
Simplified schematic of behaviors expressed by MO and drug addiction patients

Identification of early signs or symptoms of addictive behaviors in patients with MO are fundamental to break the cycle. Although it is challenging to distinguish patients with continuous or frequent headaches from MO [2, 6, 24] as headache characteristics change day-by-day [2], patients with MO generally have their headache symptoms ameliorated when medication is stopped, and withdrawal symptoms diminished [25, 26]. In addition, MO patients show behaviors such as typical fear of headache attacks - cephalalgiaphobia -, anticipatory anxiety, obsessional or ritualized drug-taking behaviors, psychological drug attachment, and abstinence symptoms after drug discontinuation [27]. The Severity of Dependence Scale (SDS) questionnaire provided by the World Health Organization (WHO) has been suggested to help in MO diagnosis [28]. Although SDS is a screening questionnaire for behavioral dependence, SDS scores are correlated with MO, and they also predicted the probability of successful withdrawal [29, 30]. A simplified summary of behaviors expressed by MO and drug addiction can be found in Fig. 1.

## Common systems in MO and drug addiction

The neuronal mechanisms of MO are not fully clear. However, some systems have been identified. Evidence from both animal and human studies suggests that MO patients have higher excitability of the nociceptive pathways during and between migraine attacks, leading to cutaneous allodynia [20]. Cutaneous allodynia reflects the central sensitization, and several studies support the role of pro-nociceptive substances in this phenomenon. Allodynia can also be caused by repeated administration of triptans, which have been demonstrated in experimental models. The chronic use of triptans can cause facilitation of descending pro-nociceptive pathways that consequently increase responsiveness to migraine triggers [67–69]. Pro-nociceptive substances such as CGRP and neuronal nitric oxide synthase (nNOS) in the dural afferent neurons of the trigeminal ganglion have shown to decrease the pain threshold that triggers migraine attacks [20]. Conversely, application of inflammatory mediators to the dura mater can cause responses to previously insensitive mechanical stimulation, additionally those mediators have also been linked to the sustained activation of trigeminal afferents [70, 71]. Previous studies assessing the effects of long-term treatments with triptans or morphine have shown an upregulation of CGRP or nNOS in the trigeminal dural afferents that persisted even after discontinuation of the treatment [20, 72, 73]. This imbalance of nociceptive substances in the dural afferent neurons has been suggested to be critical to the neuroadaptations that lower the threshold for migraine attack.

**Fig. 2:**
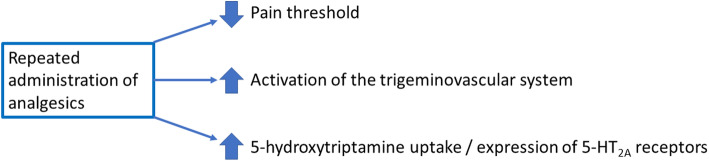
Simplified summary of changes produced by repeated administration of triptans or analgesics

Another neurotransmitter that is certainly playing a role is 5-hydroxytryptamine (5-HT, serotonin). The specific antimigraine drugs are 5-HT_1B/1D_ receptor agonists and, not surprising, the 5-HT system is dysfunctional in patients and animals chronically treated with non-specific medications, i.e. common analgesics [74]. Patients overusing analgesics have lower 5-HT levels, higher 5-HT uptake and higher 5-HT_2A_ receptor density in blood platelets [75–78]. Drug withdrawal reversed those changes, which inversely correlated with the clinical improvement. Preclinical studies, however, have demonstrated different outcomes for short- or long-term treatments with analgesics. A 15 day-acetaminophen treatment in rats produced an increase in platelet 5-HT concentration, which was accompanied by downregulation of the 5-HT_2A_ receptor and upregulation of the 5-HT transporter in the frontal cortex [79]. Conversely, a 30 day-acetaminophen treatment produced normalization of platelet 5-HT levels that correlated with reduction in analgesic effects [79]. Other studies have shown an upregulation of 5-HT_2A_ receptor in the cerebral cortex and trigeminal ganglia following prolonged administration of acetaminophen, which also correlated with an increase in frequency of cortical spreading depression and higher potentiation of trigeminal nociception. Ketanserin, a 5-HT_2A_ receptor antagonist, significantly attenuated those effects [80]. These studies suggest that, while the analgesic efficacy is correlated with an increase in platelet 5-HT levels, the normalization of platelet 5-HT levels as well as the changes in the 5-HT density after prolonged analgesic administration may be associated with increased headache frequency. A simplified summary of the effects of long-term administration of triptans or analgesics can be found in Fig. 2.

As demonstrated above, several neuroadaptations occur over the course of headache chronification and regular analgesic administration. Certain behaviors from MO patients, such as ritualized drug taking and psychological attachment to the drug also suggest that other neurotransmitter systems are involved. As we hypothesized above, DA may be playing a role in MO. DA is critical to multiple brain functions as well as it modulates reward and reinforcement. To mention a few examples, DA neurotransmission is found altered in Parkinson’s disease, depression, drug addiction, and chronic pain. Studies in chronic back pain patients using PET neuroimaging have shown alterations in affective state regulated by DA [55, 81–83], confirming its modulation to pain sensitivity and perception in patients [84, 85]. MOH is a chronic pain condition and recent genetic association studies support dopaminergic alterations in MO. A case-control study has shown that the presence of a 19-bp insertion/ deletion polymorphism (rs72393728/ rs141116007) to the dopamine-beta-hydroxylase gene correlated with the development of MO in chronic migraine patients [8, 86]. Conversely, carriers of the rs7590387GG polymorphism in the receptor activity modifying 1 (RAMP1) locus are correlated with lower risk of episodic migraine transformation to MO [87]. In drug addiction, DA levels and receptor density fluctuate during the different stages of drug use, withdrawal, and abstinence [88–90]. Specifically, DA D_2_ receptor gene (DRD2) [91–93] and the allele 9 of DA transporter (DAT) [94–97] have been associated with the susceptibility to drug abuse. DaSilva et al. (2017) demonstrated imbalance of DA D_2_/D_3_ receptors during migraine attacks and neuroimaging studies have revealed dysfunctions in the mesocorticolimbic dopamine circuit in MO patients [98]. The above evidence supports our hypothesis of altered dopaminergic circuitry in contributing either to the expression of MO or to an increase in the risk for MO, or both.

**Fig. 3:**
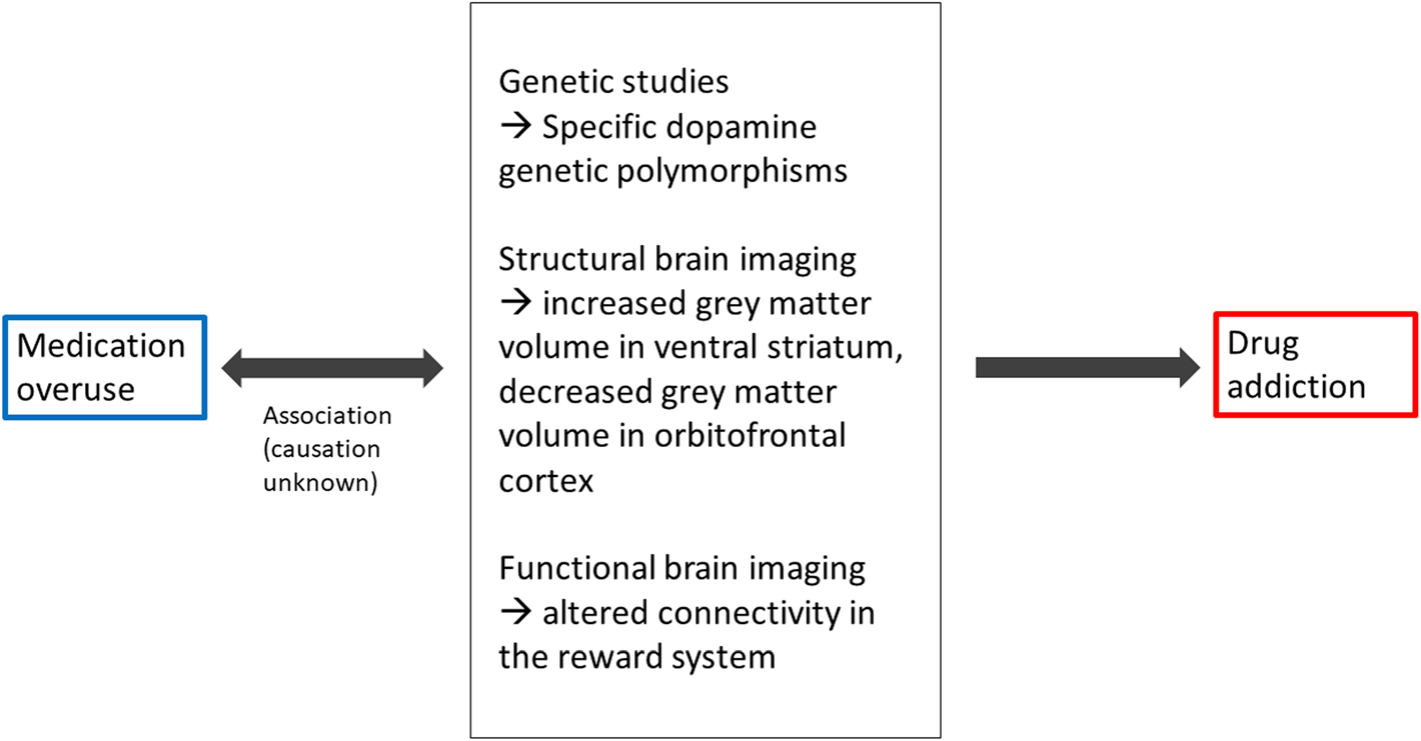
Simplified neuroimaging findings that overlap for MO and drug addiction

In human subjects, neuroimaging studies have suggested similarities between MO and drug addiction. Structural brain MRI with morphometric measurements showed that patients with MO, compared with healthy controls, had an increased grey matter volume in the ventral striatum, an area implicated in reward behaviors and addiction [99]. Furthermore, when considering patients with CM, those with MO have decreased grey matter volume in the orbitofrontal cortex compared with those without MO; this finding is relevant because orbitofrontal cortex is part of the mesocorticolimbic system implied in addictive behaviors [100]. Functional magnetic resonance imaging (fMRI) uses blood-oxygenation-level-dependent (BOLD) signal to investigate regional changes in blood oxygenation patterns, providing an indirect measurement of brain connectivity. The available fMRI studies show that patients with MO, compared with episodic migraine, have altered connectivity in regions of the pain reward system, including the nucleus accumbens, putamen, caudate, hippocampus, periaqueductal gray, precuneus, and the insula, suggesting that MO might involve the same brain areas as drug addiction [101]. A simplified summary of findings from neuroimaging studies for MO and drug addiction can be found in Fig. 3.

## Treatments of MO

While recently the combination of withdrawal and preventive medication was recommended as the most successful treatment of MOH [102], multiple studies have suggested withdrawal as the primary treatment of choice for MO [61, 103, 104]. Withdrawal does not only reduce the headache attacks, but also improve responsiveness to acute or prophylactic drugs [22, 61]. The most common symptoms experienced during withdrawal are initial worsening of headache, nausea, vomiting, hypotension, tachycardia, sleep disturbances, restlessness, anxiety, and nervousness [6, 22]. They normally last between 2 to 10 days, and do not persist longer than 4 weeks [22, 105]. Furthermore, it is important to set the correct treatment expectations for the patients, i.e. make them aware of no-full ablation of their primary headache, to achieve treatment success [106].

In most cases, withdrawal can be established by outpatient management. For instance, patients overusing triptans have shorter withdrawal symptoms when compared to ergotamine-treated or NSAIDs-treated patients [6, 22]. However, inpatient treatment should be offered to complex cases such as patients overusing opioids or barbiturates, those who show psychological problems, severe medical comorbidities, failures from previous withdrawal treatments and/or those with severe withdrawal symptoms [22, 61]. Abrupt withdrawal without tapering is advisable for patients overusing simple analgesics, ergotamine, and triptans, while tapering is recommended for those under opioids or barbiturate treatments [22, 107].

Preventive medication such as monoclonal antibodies acting on the CGRP pathway [108–111] or Onabotulinum-toxin A [61, 112] can be prescribed prior to the withdrawal treatment [113, 114]. Interdisciplinary approaches with psychological counselling and behavioral interventions are beneficial [61, 115] for complex cases [22, 61, 105]. Psychotherapy combined with short-term pharmacotherapy appears to increase the success rate in preventing relapses rather than pharmacological interventions alone, i.e. tapering medications [116].

Furthermore, patient’s engagement and behavioral interventions are both necessary for successful treatment. Interventions such as cognitive behavior therapy, management of stress, relaxation training, biofeedback, management of comorbidities, enhancement of adherence and encouragement have proved to help in the recovery [117]. Self-help groups can additionally contribute to improve coping of cephalalgiaphobia in MO patients [118].

Considering that over a quarter of MO patients relapse within the first year [60, 61], a regular follow-up of the patients is recommended to reduce the risk to relapse during remission [61]. It is also important to identify and treat comorbidities, such as mood disorders, anxiety, concurrent use of psychoactive substances, psychological dependence, and pain catastrophizing, accordingly [42, 119]. If a MO patient shows any signs of addictive behaviors a psychiatrist specialized in addiction should be engaged to initiate other relevant treatments [120]. Opioids are among the most difficult drugs to withdraw [61, 120] and, therefore, psychiatrists could provide additional assistance and support to increase the success rate and prevent relapses. From a clinical point of view, remediation at this crucial point of unstable reversibility of MO requires efficient healthcare policies that include patients with chronic migraine complicated by MO in specific public health addiction rehabilitation programs (106).

## Conclusions

Several studies support that MO shares certain behavioral, genetic, and neuronal pathways with drug addiction. DA might likely be involved in the pathophysiology of the secondary headache and though the mechanism is unclear, over the course of recurrent attacks and drug administration DA actions appear to increase in several brain regions. Consequently, DA could either lead to an increase in the risk for MO or to the manifestation of MO. Opioids prescription must be completely avoided considering its high potential of abuse and high economical and societal costs. Chronic headache patients holding similar risks to addiction should be followed up with more discretion to prevent the development of MO and addictive behaviors. Clinical interventions to those patients showing initial signs and symptoms of addiction should be performed promptly. While recently the combination of withdrawal and preventive medication was recommended as the most successful treatment of MOH, multiple studies have suggested withdrawal as the primary treatment of choice for MO. If relapse occurs repeated times and/or a patient has a complex case with addictive behaviors, a psychiatrist should be brought on board to take further measures. Combined psychological and pharmacological interventions can also increase the success rate for MO patients in remission. Further studies are still warranted to clarify the role of DA in MO, as well as to understand the molecular mechanisms of chronic use of medications in the development of the secondary headache disorder such as MO.

## Data Availability

Not applicable.

## Abbreviations

5-HT: 5-hydroxytriptamine (serotonin)
CGRP: calcitonin gene-related peptide
CM: chronic migraine
COX: cyclooxygenase
DA: dopamine
DRD2: dopamine receptor D2
GABA: gamma-aminobutyric acid
ICHD: International Classification of Headache Disorders
MO: medication overuse
MOH: medication overuse headache
nNOS: neural nitric oxide synthase
NSAIDs: non-steroidal anti-inflammatory drugs
PAG: periaqueductal grey (area)
RAMP1: receptor activity modifying protein 1
