# Correction to: Medication overuse and drug addiction: a narrative review from addiction perspective

**DOI:** 10.1186/s10194-021-01275-x

**Published:** 2021-07-05

**Authors:** Tatiane Teru Takahashi, Raffaele Ornello, Giuseppe Quatrosi, Angelo Torrente, Maria Albanese, Simone Vigneri, Martina Guglielmetti, Cristiano Maria De Marco, Camille Dutordoir, Enrico Colangeli, Matteo Fuccaro, Davide Di Lenola, Valerio Spuntarelli, Laura Pilati, Salvatore Di Marco, Annelies Van Dycke, Ramla Abuukar Abdullahi, Antoinette Maassen van den Brink, Paolo Martelletti, on behalf of the European Headache Federation School of Advanced Studies (EHF-SAS)

**Affiliations:** 1 Headache Research, Wolfson CARD, Institute of Psychiatry, Psychology & Neuroscience, King’s College London, 20 Newcomen St, London, SE1 1YR UK; 2 Present address: Medicines Discovery Catapult, Block 35, Mereside, Alderley Park, Cheshire, SK10 4TG UK; 3 Neuroscience Section, Department of Applied Clinical Sciences and Biotechnology, University of L’Aquila, Via Vetoio 1, Coppito, 67100 L’Aquila, Italy; 4 Department of Health Promotion, Mother and Child Care, Internal Medicine and Medical Specialties (PROMISE), University of Palermo, Piazza delle Cliniche 2, 90127 Palermo, Italy; 5 Department of Biomedicine, Neurosciences and Diagnostic (Bi.N.D.), University of Palermo, Via del Vespro 129, 90127 Palermo, Italy; 6 Department of Systems Medicine, University of Rome “Tor Vergata”; Neurology Unit, “Tor Vergata” Hospital, Viale Oxford, 81, 00133 Rome, Italy; 7 Department of Experimental Biomedicine and Clinical Neurosciences, University of Palermo, Via del Vespro, 129, 90127 Palermo, Italy; 8 Pain Medicine Unit, Santa Maria Maddalena Hospital, Occhiobello, Italy; 9 Regional Headache Referral Center, Sant’Andrea Hospital, Via di Grottarossa 1039, 00189 Rome, Italy; 10 Department of Medical, Surgical and Experimental Sciences, University of Sassari, Piazza Università, 21, 07100 Sassari, Italy; 11 Department of Neurology, University Hospital Ghent, Corneel Heymanslaan 10, 9000 Ghent, Belgium; 12 Department of Neurology, Conegliano Hospital, Via Brigata Bisagno, 2, 31015 Conegliano, Italy; 13 Department of Medico-Surgical Sciences and Biotechnologies, “Sapienza” University of Rome, Polo Pontino, Viale XXIV Maggio 7, 04100 Latina, Italy; 14 Department of Internal Medicine, Division of Pharmacology, Erasmus MC University Medical Centre Rotterdam, Wytemaweg 80, 3015, CN Rotterdam, The Netherlands; 15 Department of Neurology, AZ Sint-Jan Brugge, Ruddershove 10, 8000 Brugge, Belgium; 16 Headache Centre, Guy’s and St Thomas NHS Trust, London, UK; 17 Department of Clinical and Molecular Medicine, Sapienza University of Rome, Via di Grottarossa 1039, 00189 Rome, Italy

**Correction to: J Headache Pain 22, 32 (2021)**

**https://doi.org/10.1186/s10194-021-01224-8**

Following the publication of the original article [1], we were notified that the correct affiliation for Dr. Annelies Van Dycke is:

Department of Neurology, AZ Sint-Jan Brugge, Ruddershove 10, 8000 Brugge, Belgium

Also the acronym for the European Headache Federation School of Advanced should be EHF-SAS.

The original article has been corrected.
